# Assessment of IL-8, RANTES, MIG, MCP-1, IP-10, and IL-12p70 and Their Association with Anxiety and Quality of Life in Patients with Chronic Kidney Disease or After Kidney Transplantation

**DOI:** 10.3390/ijms252413449

**Published:** 2024-12-15

**Authors:** Gabriela Furtak, Natalia Lerch, Mateusz Kozłowski, Piotr Tkacz, Emilia Piekara, Maria Łagódka, Damian Durys, Izabela Gutowska, Krystyna Widecka, Małgorzata Marchelek-Myśliwiec, Wojciech Żwierełło, Aneta Cymbaluk-Płoska

**Affiliations:** 1 Department of Reconstructive Surgery and Gynecological Oncology, Pomeranian Medical University in Szczecin, Powstańców Wielkopolskich 72, 70-111 Szczecin, Poland; 2 Department of Biochemistry and Medical Chemistry, Pomeranian Medical University in Szczecin, Powstańców Wielkopolskich 72, 70-111 Szczecin, Poland; 3 Department of Hypertension and Internal Medicine, Pomeranian Medical University in Szczecin, Unii Lubelskiej 1, 71-252 Szczecin, Poland; 4 Clinical Department Nephrology, Transplantology & Internal Medicine, Pomeranian Medical University in Szczecin, Powstańców Wielkopolskich 72, 70-111 Szczecin, Poland

**Keywords:** anxiety, quality of life, STAI, SF-36, logit, CKD, KTx, RANTES, IL-8, MIG

## Abstract

Anxiety is a subjective feeling of fear in response to stressful or threatening situations. Chronic diseases (such as chronic kidney disease) or the state after kidney transplantation are such situations and they may result in a decreased quality of life. The main aim of this research was to evaluate if the proteins IL-8, RANTES, MIG, MCP-1, IP-10, and IL-12p70 could be indicators of higher levels of anxiety or decreased quality of life in chronically ill women. The assessment was conducted using the STAI and SF-36 questionnaires and with the measurement of listed proteins from the patient’s blood sample. The study group consisted of 107 women—101 patients from the Nephrological Clinic and 6 from the Dialysis Center. Both of the facilities are part of University Clinical Hospital No. 2 in Szczecin. Statistical analysis was performed using JASP software (JASP 0.18.3 version). Anxiety levels and quality of life correlations between STAI and SF-36 scores and individual variables were assessed. Logistic regression models were performed for both questionnaire outcomes: SF-36 and STAI. Lower quality of life was found in the group with a higher IL-8 concentration compared to the group of women with a lower IL-8 concentration. A positive weak correlation was found between a decreased quality of life and IL-8 and RANTES. A higher RANTES level increases the odds of a lower quality of life. This study shows that special care should be provided for chronically ill women (especially with CKD or after KTx) with a higher RANTES or IL-8 concentration. They would highly benefit from close monitoring of their mental health.

## 1. Introduction

Anxiety is described as a subjective feeling of fear in response to stressful or threatening situations [1]. It is a disturbing experience, often defined as waiting for an imminent danger [2]. Anxiety may appear as an adaptive survival feature, but when it becomes persistent and overwhelming, it is already classified as an anxiety disorder [1,2]. Although anxiety functions as a basic emotion, it can be a symptom of many diseases, such as depression [3]. Moreover, anxiety can be distinguished as a stable personality trait, characterized by a tendency to respond with anxiety to specific situations, or as a transient emotional state [4]. A widely used tool for assessing both temporary anxiety (anxiety as a state) and long-term levels of anxiety (anxiety as a trait) is the State-Trait Anxiety Inventory (STAI) [5]. Besides anxiety itself, health-related quality of life is also assessed by the SF-36 questionnaire (Short Form Health Survey), covering eight aspects of health, such as physical and social functioning along with limitations associated with these, mental health, pain, and overall perception of health [6]. In their studies of human behavior, researchers measure not only fear but also management mechanisms, for example, using the Coping Orientation to Problems Experienced (COPE) scale [7,8]. Neurobiological processes such as neurogenesis, synaptic transmission, and neuron-glia communication, involving chemokines, are crucial factors in mental disorders [9,10]. The activation of the inflammatory response and the prolonged dysregulation of several axes disrupts chemokine functions, leading to mental disorders, including anxiety and depression [11]. Interleukin-8 (IL-8) is a pro-inflammatory cytokine, which is produced by macrophages and other mesenchymal cells including microglia [12]. It acts as a chemotactic agent for neutrophils and lymphocytes in inflammatory activity [13]. IL-8 is a chemokine that has neuroprotective properties in addition to its role in the immune system. Its effect on neuropsychiatric disorders is not fully understood, but recent studies have found that IL-8 could be implied in the pathogenesis of psychiatric disorders [12,14]. Furthermore, it was proposed that IL-8 played a role in the molecular processes that mediated anxiety resilience [14]. Monokine induced by interferon gamma (MIG) is a cytokine in the CXC family, produced by macrophages and fibroblasts in response to gamma interferon stimulation [15]. It plays a crucial role in the recruitment of granulocytes and mononuclear cells, contributing to the maintenance of inflammation [16]. This protein is associated with early function and acute transplant rejection [17]. Multiple studies indicate a substantial correlation between depression and IFN-γ [11]. Monocyte chemoattractant protein-1(MCP-1), often called ligand 2 (CCL2), belongs to the chemokine group CC [18]. MCP-1 is involved in increasing the immune response to Th1 and Th2 [19]. It participates in inflammatory processes and acts as a chemoattractant for cells and other pro-inflammatory cytokines. CCL2/MCP-1 has been linked to anxiety and depression [20]. Many chronic diseases are directly or indirectly associated with MCP-1 [18]. Regulated upon Activation, Normal T Cell Expressed and Secreted (RANTES) are inductors of monocytes, T cells, and NK cells to places where inflammation and infection occur [21]. RANTES, also known as chemokine CCL5, belong to the CC motif chemokine family and with its receptor CCR5 facilitate the inflammatory response [22]. Recent studies have shown the protumorigenic role of the CCL5/CCR5 axis due to its abilities in angiogenesis and metabolic reprogramming [23,24]. Since research on this specific chemokine has only recently began, there are scarce data on CCL5. However, some of them associated CLL5 with anxiety and depression [11]. Interferon gamma-induced protein 10 (IP-10), also known as CXCL10, is a chemokine produced mainly by monocytes [25]. Together with the CXCR3 receptor, it is involved in the pathogenesis of many autoimmune diseases [26]. It also has anti-tumor effects, due to its anti-angiogenic functions. Studies on CXCL10 have shown that its urinary concentration is more sensitive than creatinine levels and can serve as a biomarker for detecting nephritis, including systemic lupus erythematosus [27]. The chemokine level was also increased in a significant way in patients who exhibited high levels of anxiety, as well as depressive symptoms [28]. IL-12p70 is an active heterodimer of IL-12, composed of two subunits, p35 and p40. The p40 subunit can function independently or as a homodimer, while the p35 subunit requires binding with p40 [29]. IL-12p70 is involved in the differentiation of T cells in Th1 and regulates the activity of NK cells, supporting the immune system’s response [29,30]. The effects of IL-12 on T and NK cells have been shown in the past, with a focus on defining its role in stimulating the production of IFN-γ during infection and inflammation [31]. In addition, it shows antiangiogenic properties [30]. Moreover, previous studies linked psychiatric morbidity (anxiety disorders or depression) with level of IL-12 [32]. Without a doubt, chronically ill patients are a group at a higher risk of developing symptoms and anxiety disorders. This is mainly due to increased stress, uncertainty about the future, and frequent medical visits [33]. The prolonged anxiety that accompanies this group of patients can lead to a deterioration of treatment results, decreased quality of life, and the onset of other mental disorders such as depression [34]. Therefore, in our study, we additionally analyzed the levels of selected proteins in the serum of female patients, attempting to find a correlation between anxiety levels (assessed using the STAI and SF36) and the concentration of these proteins. Identifying potential biomarkers for managing anxiety and its levels would be particularly important for chronically ill patients, allowing for more precise diagnosis and treatment of these conditions. The aim of our study was to measure anxiety levels and quality of life among women suffering from chronic diseases such as CKD and women after kidney transplantation in Szczecin, using the STAI and SF-36 questionnaire and their association with IL-8, RANTES, MIG, MCP-1, IP-10 and IL-12p70. The following hypotheses were posed:

1. Higher anxiety correlates with higher levels of IL-8, RANTES, MIG, MCP-1, IP-10 and IL-12p70.
2. Lower quality of life correlates with higher levels of IL-8, RANTES, MIG, MCP-1, IP-10 and IL-12p70.
3. If the woman has a higher concentration of IL-8, RANTES, MIG, MCP-1, IP-10 or IL-12p70 then the odds of an increased anxiety level increase.
4. If the woman has a higher concentration of IL-8, RANTES, MIG, MCP-1, IP-10 or IL-12p70 then the odds of a decreased quality of life increase.

## 2. Results

### 2.1. Group Characteristics

To confirm the scale reliability of the research results, which were acquired utilizing the SF-36 and STAI questionnaires, Cronbach’s alpha coefficient and split-half reliability were used.

Both parts—s-STAI and t-STAI, as well as the STAI questionnaire as a whole—had an acceptable internal consistency of 0.923, 0.921, and 0.954, respectively. The split-half reliability coefficient was 0.869. The halves strongly correlated with each other. As for the correlation coefficient—its value was 0.769. According to questionnaire results, 23/107 patients had high total anxiety, 15/107 women had high anxiety as a state, and 34/107 had high anxiety as a trait.

Cronbach’s alpha coefficient of the SF-36 questionnaire was 0.866. The split-half reliability coefficient for SF-36 was 0.929 and the halves strongly correlated with each other. The correlation coefficient was 0.892.

Weak left-sided asymmetry characterized the age distribution. The basic statistics of the women participating in the study are included in Table 1.

Women were treated by hemodialysis or KTx (Table 1). Among them, eight women had two kidney transplants and one had three. The age of the last transplant was reported. The minimum age of the transplant was 1 year, the maximal was 21 years. The average age of the kidney transplant was 9.65 years and the median was 10 years. Over one-third of the patients had diabetes and over two-thirds of them had diabetes induced by transplantation (Table 1). Other cases were DM1 or DM2.

Minimal GFR was 4 mL/min/1.73 m^2^, maximal 105 mL/min/1.73 m^2^, average GFR was 47 mL/min/1.73 m^2^ and median was 48. Table 2 contains more detailed statistics about the patient’s disease and treatment.

Among the women with an active kidney transplant, the largest group with known CKD, the reason was they were the group of patients with glomerulonephritis (GN). Other reasons were ADPKD, diabetic kidney disease, and hypertensive kidney disease. Other patients had CKD for unknown reasons (Table 2). Patients with a kidney transplant were treated with tacrolimus (TACR), with the average concentration of 6.77 ng/mL, mycophenolate mofetil (MMF), encorton (ENC), and cyclosporin A (CSA) with an average concentration of 95.95 ng/mL, everolimus (EVE), azathioprine (AZA), and sirolimus (SIR) (Table 2).

Basic statistics on scores from questionnaires taken by our patients are described in Table 3.

Basic statistics on the concentration of the studied protein in our patients are described in Table 4.

### 2.2. Comparison of STAI, SF-36 Scores, IL-8, RANTES, MIG, MCP-1, IP-10, IL-12p70, and Individual Variables in the Study Subgroups

#### 2.2.1. Studied Proteins

In order to compare the scores from the questionnaires in patients with higher concentrations of proteins to those with lower concentrations, we divided all patients into two groups based on their protein concentration. Each time, the dividing level assumed the value of the median concentration of a particular protein, which means that a patient was ranked to a group with a higher concentration of that particular protein, if patients had at least 14.335 ρg/mL in case of dividing by IL-8 concentration, 1916.95 ρg/mL for RANTES, 91.83 ρg/mL for MIG, 15.95 ρg/mL for MCP-1, 81.85 ρg/mL for IP-10, and 3.53 ρg/mL for IL-12p70.

Testing the distributions of SF-36 scores proved that the lower IL-8 concentration group had lower SF-36 scores (*p* = 0.033), which was tested in a one-sided MWU test. In the one-sided MWU test for RANTES protein the alternative hypothesis implies that the lower RANTES concentration group had lower SF-36 scores in comparison with the higher RANTES concentration group (*p* = 0.028). Other combinations of comparison tests of s-STAI, t-STAI, STAI-total, SF-36, and IL-8, RANTES, MIG, MCP-1, IP-10, and IL-12p70 in one-sided and two-sided tests did not prove their significance. Tested with the Bonferroni method, none of the abovementioned results proved its significance.

#### 2.2.2. SF-36

With the aim of comparing the parameters of the patients with higher and lower quality of life, we divided patients by SF-36 scores and qualified the patients with at least 65 points (value of median) to group 1 due to higher SF-36 scores. The rest of the women were qualified to group 2.

Significant differences were found in the distributions of IL-8 (*p* = 0.004) and RANTES (*p* = 0.020), and s-STAI, t-STAI, and STAI-total (all of those three have *p* < 0.001) scores for the groups of higher and lower SF-36 scores. Applying the correction for multiple comparisons, the Bonferroni correction was used and proved the statistical significance of the IL-8 outcome and s-STAI, t-STAI, and STAI-total.

Other alternative hypotheses claim that the group with lower SF-36 scores has lower IL-8 (*p* = 0.002) and RANTES (*p* = 0.010), and s-STAI, t-STAI, and STAI-total (all of those three have *p* < 0.001) scores compared to the group of higher SF-36 scores, were also positively verified. The Bonferroni method that was used additionally revealed the significance of IL-8 and s-STAI, t-STAI, and STAI-total.

#### 2.2.3. STAI

For the purposes of the comparison of this analysis, STAI scores that were quantitative data were changed to nominal data. STAI-total was divided into two groups: first with higher scores of at least 94 and second lower than 94. Groups for s-STAI and t-STAI were divided into those with at least 46 and under 46.

Groups of patients with higher and lower scores were compared in terms of protein concentration. The significant difference in STAI-total scores between both groups was found only in RANTES concentration (*p* = 0.033). Testing the one-sided hypothesis, the alternative hypothesis that a group with lower STAI-total scores had lower RANTES concentration (*p* = 0.017) was proved. Tested with the Bonferroni method, none of the abovementioned results proved its significance.

The distributions of RANTES concentration by the level of s-STAI scores showed no significant differences (*p* = 0.073). When the alternative hypothesis was changed and the one-sided hypothesis test was carried out, the groups turned out to be not equal (*p* = 0.037). The H1 was proved as the group of patients with lower s-STAI had lower RANTES concentration in comparison to the group with higher s-STAI scores. However, this did not prove its significance while testing with the Bonferroni method.

A group with a lower s-STAI score had a higher GFR compared to a group with a higher s-STAI score (*p* = 0.029).

When testing other hypotheses with s-STAI, t-STAI, and STAI-total and IL-8, RANTES, MIG, MCP-1, IP-10, IL-12p70, and GFR, no significant differences were proven.

#### 2.2.4. GFR

With the objective of comparing questionnaire outcomes and protein concentrations of groups with higher and lower GFR, we divided the women into two groups according to their GFR value (under 45 mL/min./1.73 m^2^ and at least 45 mL/min./1.73 m^2^).

Between the two groups there were significant differences in the concentration of IL-8 (*p* = 0.006), MIG (<0.001), MCP-1 (*p* = 0.004), and IP-10 (*p* = 0.017). There were no significant differences in the case of RANTES (*p* = 0.967) nor IL-12p70 (*p* = 0.467). The difference between groups was significant in s-STAI values (*p* = 0.017) but insignificant in SF-36 (*p* = 0.174), t-STAI (*p* = 0.699), and STAI-total (*p* = 0.206) values.

Furthermore, in a group of lower GFR there were found to be significantly higher concentrations of IL-8 (*p* = 0.003), MIG (<0.001), MCP-1 (*p* = 0.002), and IP-10 (*p* = 0.09). There was also a higher s-STAI score (*p* = 0.008). This alternative hypothesis did not prove its significance in the case of RANTES (*p* = 0.519) nor IL-12p70 (*p* = 0.233), as well as in SF-36 (*p* = 0.087), t-STAI (*p* = 0.350), and STAI-total (*p* = 0.206) values.

#### 2.2.5. Diabetes

The distributions of IL-8, RANTES, MIG, MCP-1, IP-10, and IL-12p70 scores by the presence of diabetes diagnosis showed no significant differences, relatively (*p* = 0.341), (*p* = 0.324), (*p* = 0.076), (*p* = 0.866), (*p* = 0.156) and (*p* = 0.668).

Testing the alternative hypotheses—one was proved to be statistically significant and was positively verified in a group of women without diabetes as MIG concentration was higher (*p* = 0.038) compared to a group with diabetes.

The distribution of SF-36 scores in patients with and without diabetes showed no significant difference (*p* = 0.226).

Likewise, the distribution of all STAI scores showed no significant difference when comparing groups with diabetes and without diabetes.

#### 2.2.6. Immunosuppressants

In an attempt to provide a more accurate understanding of the interactions between inflammation, anxiety, and quality of life and to control the possible influence of the potential confounding variable, immunosuppressants, which may have influenced the levels of inflammatory cytokines and psychological outcomes, we compared a group of patients treated with the given drug with a group of patients that did not use this drug.

There were significant differences between both groups of patients divided based on tacrolimus treatment regarding SF-36 (*p* = 0.004), IL-8 (*p* = 0.023), RANTES (*p* = 0.028), MIG (*p* = 0.045), and IP-10 (*p* = 0.038). There were no significant differences in s-STAI, t-STAI, STAI-total, MCP-1, IL-12p70, and GFR.

There were no significant differences between both groups of patients divided based on mycophenolate mofetil (MMF) treatment as well as sirolimus (Sir) treatment regarding SF-36, s-STAI, t-STAI, STAI-total, IL-8, RANTES, MIG, MCP-1, IP-10, and IL-12p70. There were only significant differences in GFR (*p* < 0.001—MMF and *p* = 0.041—Sir).

There were significant differences between both groups of patients divided based on azathioprine (AZA) treatment regarding s-STAI (*p* = 0.020) and SF-36 (*p* = 0.039). There were no significant differences regarding other variables.

There were no significant differences between both groups of women divided based on prednisolone, everolimus, or cyclosporin treatment.

There was a significant difference between patients treated with immunosuppressants compared to those with no such treatment regarding MCP-1 (*p* = 0.003) and IL-8, MIG, IP-10, and GFR (*p* < 0.001 for the four abovementioned).

Analyses of these results should be cautious, taking into consideration that the number of patients treated with the given immunosuppressants were as follows: Rapa/Sir—2, AZA—8, Everolimus—8, CsA—12 and prednisolone—60, MMF—68, and tacrolimus—77. The number of patients treated with no immunosuppressants was six.

### 2.3. Correlations Between STAI, SF-36 Scores, IL-8, RANTES, MIG, MCP-1, IP-10, IL-12p70 and Individual Variables

In the undermentioned analysis of correlation, the following degrees of correlation were applied: 0 indicates no relationship, values over 0 to ±0.3 implies a weak degree of correlation, over ±0.3 to ±0.5 implies moderate, over ±0.5 to ±1 implies strong, and ±1 is indicative of perfect correlation. All of the analyzed correlations were presented in the correlation table (Table 5). The analysis revealed a significant and strong correlation between s-STAI and t-STAI scores among all women (rs = 0.769). The detected correlation was in the positive direction, which means that when the level of anxiety as a state increased then the level of anxiety as a trait also increased in the studied population. A very strong correlation between STAI-total and s-STAI (rs = 0.934) as well as STAI-total and t-STAI (rs = 0.940) was noted.

A significant moderate and strong correlation was found between SF-36 and all STAI scores—s-STAI, t-STAI, STAI-total (rs = 0.594, rs = 0.498, and rs = 0.579 relatively). There was also a significant weak correlation in the positive direction between SF-36 and the age of patients (rs = 0.277).

Correlations between all STAI scores and studied proteins: IL-8, RANTES, MIG, MCP-1, IP-10, and IL-12p70 were nonsignificant and very weak.

Correlations between SF-36 scores and IL-8 (rs = 0.310) and RANTES (rs = 0.241) were significant and moderate and weak, respectively. The correlation between SF-36 and the other examined proteins was nonsignificant.

The correlation between age and proteins MIG (rs = 0.423), IL-8 (rs = 0.372), and IP-10 (rs = 0.305) were moderate and significant. The correlation between age and MCP-1 (rs = 0.223) was weak and significant, but the rest were not significant.

The age of kidney transplant was negatively correlated with MCP-1 (rs = −0.212). The correlation was weak and statistically significant.

There was also a significant negative moderate correlation between GFR and MIG (rs = −0.443), MCP-1 (rs = −0.365), and IL-8 (rs= −0.337), and was significant negative weak for IP-10 (rs= −0.295). In regard to RANTES and IL-12p70, the correlation was not significant and very weak. The correlation of GFR and s-STAI, t-STAI, STAI-total, and SF-36 was not significant.

IL-8 had a positive correlation with every other studied protein, moderate significance with MIG (rs = 0.489), MCP-1 (rs = 0.480), IP-10 (rs = 0.348), and IL-12p70 (rs = 0.330) and a weak significance with RANTES (rs = 0.237).

A strong positive significant correlation was observed between MIG and IP-10 (rs = 0.693) and moderate positive significance between MIG and MCP-1 (rs = 0.454) and between MCP-1 and IP-10 (rs = 0.431).

In the range of 3.5–13 [ng/mL] there was no significant correlation between tacrolimus concentration and the proteins, s-STAI, t-STAI, STAI-total, SF-36 and GFR.

### 2.4. Parameter Evaluations for Logistic Regression Models

The study’s next stage involved identifying the factors that had a substantial impact on the likelihood of experiencing more anxiety and lower quality of life. Logistic regression models were created with the following collection of independent variables in order to achieve this goal:

- age (“1” for more than 60 years old and “0” for those under 60),
- diabetes (“1” for women with diabetes and “0” for women without diabetes)
- GFR (“1” for higher than 45 and “0” for 45 or lower)
- IL-8 (“1” for a minimum of 14.335 ρg/mL and “0” for lower values)
- RANTES (“1” for a minimum of 1916.95 ρg/mL and “0” for lower values)
- MIG (“1” for a minimum of 91.83 ρg/mL and “0” for lower values)
- MCP-1 (“1” for a minimum of 15.95 ρg/mL and “0” for lower values)
- IP-10 (“1” for a minimum of 81.85 ρg/mL and “0” for lower values)
- IL-12p70 (“1” for a minimum of 3.53 ρg/mL and “0” for lower values)
- Tacrolimus (“1” for patients treated with this drug and “0” for not using this drug)
- Mycophenolate mofetil (“1” for patients treated with this drug and “0” for not using this drug)
- Prednisone (“1” for patients treated with this drug and “0” for not using this drug)
- Azathioprine (“1” for patients treated with this drug and “0” for not using this drug)
- Sirolimus (“1” for patients treated with this drug and “0” for not using this drug)
- Everolimus (“1” for patients treated with this drug and “0” for not using this drug)
- Cyclosporin (“1” for patients treated with this drug and “0” for not using this drug)

As dependent variables, the following were accepted:

- SF-36 (“1” for at least 65 points and “0” for lower scores)
- STAI-total (“1” for at least 94 points and “0” for lower scores),
- s-STAI (“1” for at least 46 points and “0” for lower scores),
- t-STAI (“1” for at least 46 points and “0” for lower scores).

With the aim of finding the best combination of variables that would affect the probability of higher anxiety or lower quality of life in a significant way, a formal selection of variables was conducted for each logistic regression model, using logistic regression with the enter method, based on theoretical presumptions and statistical tests performed and described above.

#### 2.4.1. Logistic Regression Model for the Dependent Variable SF-36

The obtained logit model proved to be statistically significant (*p* = 0.001) in holistic assessment. This model explains 23.79% of the variance of our model (Nagelkerke R^2^ = 0.266). Dichotomous variables RANTES and IL-8, and GFR, age, tacrolimus, azathioprine, and sirolimus were selected as factors. Table 6 shows parameter estimates for the dependent variable SF-36 of the logistic regression model including variables that were obtained using the enter regression method.

In this model, RANTES had a positive, statistically significant impact on the dependent variable SF-36 (*p* = 0.039)—if a person had a higher concentration of RANTES then the odds of increasing SF-36 increased. TAC had a negative, statistically significant impact on the dependent variable SF-36 (*p* = 0.018)—if a woman was treated with tacrolimus, then the odds of increasing SF-36 decreased. Other variables interpreted separately did not prove to be statistically significant.

By interpreting the odds ratios (with the assumption that the other variables, which were included in the model, remain unchanged), it could be concluded that if the women had a higher RANTES level, then the chance of increasing SF-36 increased by 150%. Moreover, if the woman was treated with tacrolimus, then the chance of increasing SF-36 decreased by 73%.

Sensitivity −61.1% and specificity −71.7% of the model was also assessed and displayed in Table 7.

The estimated model’s validity was assessed. It was evaluated by counting the classification accuracy of women in the confusion matrix (Table 8).

The classification accuracy was assessed using the confusion matrix and the *R*^2^_*count*_ coefficient and also the ROC curve. The *R*^2^_*count*_ coefficient took a value of 66.355%. This was greater than 50%, so the conclusion that the classification based on the model was better than random could be obtained.

**Figure 1:**
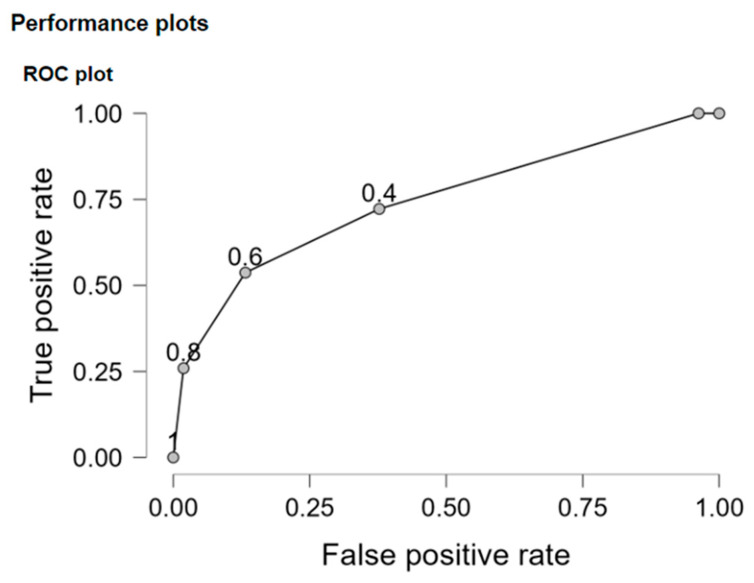
ROC curve.

The area under the ROC curve for the estimated model was equal to 0.740 (Figure 1). This was significantly greater than 0.5 (for *p* = 0.0000).

To test for multicollinearity among independent variables to ensure the logistic models are not compromised, VIF (variance inflation factor) was calculated (Table 9). The values of VIF testify that there is no or almost no multicollinearity burden for this model.

#### 2.4.2. Logistic Regression Model for the Dependent Variable STAI-Total

The parameter estimates of this logistic regression model for the dependent variable STAI-total, including variables selected with the enter regression method, were presented in Table 10. RANTES, age, and GFR were used in a continuous data set version as covariates in this model and cyclosporin (CsA), azathioprine (AZA), tacrolimus (TAC), mycophenolate mofetil (MMF), and prednisolone (PRED) as nominal factors. This logit model proved to be significant (*p* = 0.037) with the Nagelkerke R^2^ = 0.219. None of the other variables proved to be significant.

RANTES and AZA proved to have a statistically significant and positive impact on the dependent variable STAI-total, in this model. If a woman had a higher RANTES score or if a woman was treated with azathioprine, then the odds of increasing STAI-total increased. However, if a patient had a higher RANTES score, then the chances of increasing STAI-total increased by 0.1%. If a woman was treated with azathioprine, then the odds of increasing STAI-total increased over elevenfold.

Sensitivity −17.4% and specificity −98.8% of the model were assessed and displayed in Table 11.

Table 12 shows the counted classification accuracy of patients, which was used to assess the validity of the estimated model.

The validation of classification accuracy was tested by calculating the *R*^2^_*count*_ coefficient as well as the ROC curve. The *R*^2^_*count*_ coefficient was 81.308% and was greater than 50%. Hence, it was possible to obtain the conclusion that model-based classification was preferable to random.

**Figure 2:**
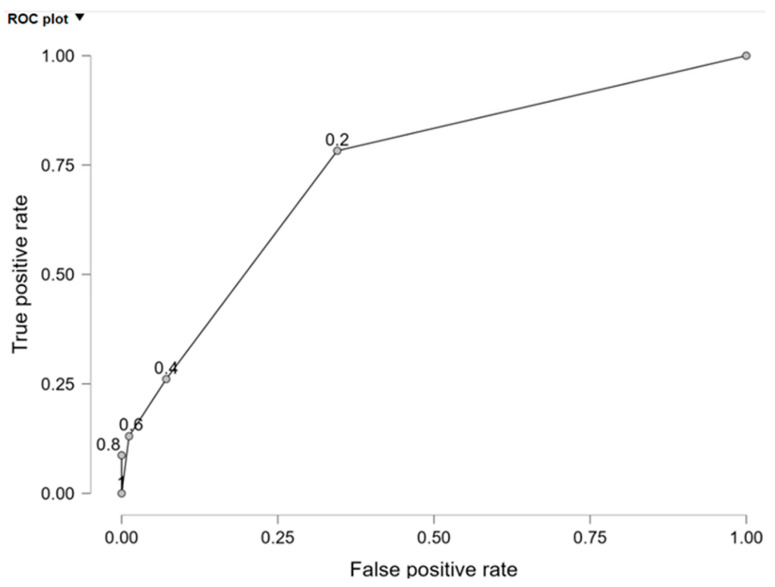
ROC curve.

The area under the ROC curve was 0.751 for the estimated model (Figure 2), significantly greater than 0.5 (for *p* = 0.0000).

To test for multicollinearity among independent variables to ensure the logistic models are not compromised, VIF (variance inflation factor) was calculated (Table 13). The values of VIF testify that there is almost no to moderate multicollinearity between variables in this model.

#### 2.4.3. Logistic Regression Model for the Dependent Variable s-STAI

The logistic regression model parameter estimates for the s-STAI (dependent variable), including variables selected using enter regression (Table 14). RANTES, MIG, age, and GFR were used in a continuous data set version as covariates in this model and TAC, MMF, AZA, and prednisolone(PRED) as nominal factors. This logit model proved to be significant (*p* = 0.022) with the Nagelkerke R^2^ = 0.277. None of the other variables tested proved to be statistically significant and when added resulted in a statistically nonsignificant model.

In this model, none of the parameters separately proved to be statistically significant.

Sensitivity −26.7% and specificity −97.8% of the model were assessed and displayed on Table 15.

The validity of the estimated model was assessed by counting the accuracy of the classification of individuals (Table 16).

Using both the *R*^2^_*count*_ coefficient as well as the ROC curve, the classification accuracy was assessed. The *R*^2^_*count*_ coefficient was 87.85% and was greater than 50%; therefore, the conclusion that the classification based on this model was superior to random, could be drawn.

**Figure 3:**
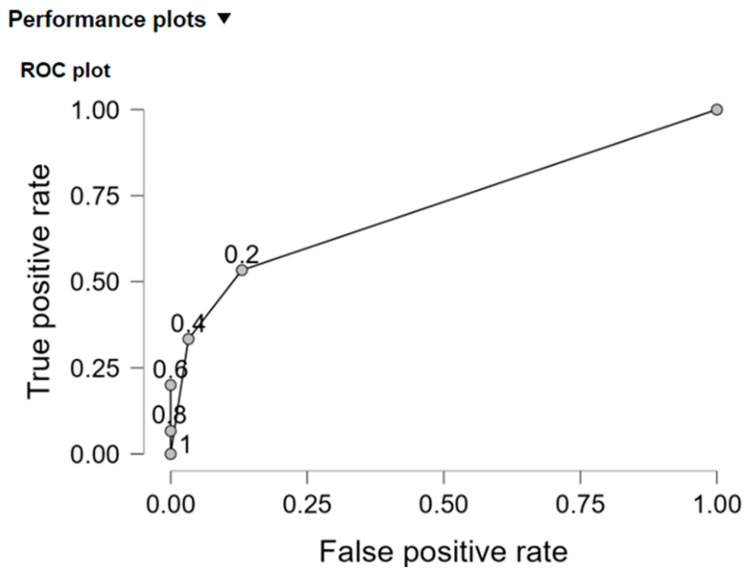
ROC curve.

The area under the ROC curve for the estimated model was 0.771 (Figure 3), which was greater than 0.5 in a significant way (for *p* = 0.0000).

To test for multicollinearity among independent variables to ensure the logistic models are not compromised, VIF (variance inflation factor) was calculated (Table 17). The values of VIF testify that there was almost no to moderate multicollinearity between variables in this model.

#### 2.4.4. Logit Model for the Dependent Variable t-STAI

None of the tested logit models proved to be statistically significant. Methods of regression: Enter, Backward, Forward, and Stepwise were used. All of the variables IL-8, RANTES, MIG, MCP-1, IP-10, and IL-12p70, as well as age, GFR, diabetes, and all of the immunosuppressants were tested.

### 2.5. Power Analysis

Under the assumptions of power 0.8, 2 sided α coefficient 0.05, and with the aim to prove correlations that were estimated at 0.25–0.3 (weak correlations), a sample size of 85–123 women would be required.

## 3. Discussion

Patients suffering from chronic kidney disease (CKD) and those who have undergone organ transplantation are exposed to significant stresses, not only physical but also psychological, which greatly impact their levels of anxiety and quality of life. Anxiety and anxiety disorders accompany patients with chronic kidney disease, not only in the terminal stage of the disease but also at earlier stages [35,36]. A significant contribution to these disorders is attributed to the necessity of regular, time consuming, and often physically exhausting dialysis procedures. The quality of life for these patients is also affected by numerous dietary restrictions and the constant need to monitor their health parameters, significantly limiting their freedom of life [37,38,39]. The group of patients also affected by anxiety states are patients after organ transplantation. This results from fears of transplant rejection and the consequent need to take immunosuppressive drugs, which carry many side effects, including a significantly increased risk of infections [36]. In recent years, research has highlighted the pivotal role of inflammation and immune system dysregulation in psychiatric conditions like anxiety and depression. Pro-inflammatory cytokines have been implicated in the development of mood disorders, including anxiety and depression, consequently reducing the patient’s quality of life [40,41]. Chronic inflammation, often present in CKD and post-transplant patients, leads to elevated levels of these cytokines, contributing not only to the physical symptoms of the disease but also to psychological distress [42]. This association between inflammatory cytokines and mood disturbances suggests that an immune-inflammatory response may partly drive the psychological symptoms experienced by CKD and transplant patients. These same inflammatory pathways, activated by chronic illness, could also exacerbate anxiety and diminish the quality of life in CKD and transplant populations, creating a vicious cycle where the disease contributes to psychological distress, which in turn worsens health outcomes [11]. The present study involved assessing anxiety as well as health-related quality of life in patients with CKD and post-transplant patients. Additionally, the correlation between elevated serum protein levels and increased anxiety levels, as well as decreased quality of life, was evaluated. To our knowledge, this is one of the few studies assessing proteins to identify potential anxiety biomarkers. In our study, the median scores of STAI, s-STAI, and t-STAI were lower in the group of patients with a higher quality of life, which means they have lower levels of anxiety in general, anxiety as a state, and anxiety as a trait than women with a lower quality of life. Moreover, a significant strong and moderate correlation was found between the quality of life and all types of anxiety. Yarlioglu et al. investigated the relationship between quality of life, anxiety, depression and the stage of CKD in ADPKD (which stands for autosomal dominant polycystic kidney disease). Their findings clearly showed that as CKD progressed, patients’ quality of life decreased while depression levels and anxiety increased [39]. Alwhaibi et al. studied the relationships between depression, anxiety, and health-related quality of life among patients with migraine. Their data indicated that individuals with higher anxiety levels had a significantly lower quality of life [43]. These studies confirm that the level of anxiety in patients is strongly correlated with how they perceive their health-related quality of life. Patients with CKD are particularly vulnerable to anxiety disorders. Our study showed a relationship between the s-STAI results and the GFR. A group with a lower s-STAI score has a higher GFR (*p* = 0.029). Similar conclusions were reached by Dziubek et al., who assessed depression and anxiety in patients with CKD and after kidney transplantation. They utilized the STAI questionnaire and found that the state anxiety level (s-STAI) was the highest in the entire study group, constituting 24%, while the trait anxiety level (t-STAI) was 18% among the respondents [35]. However, the correlation between GFR and any type of anxiety and quality of life in our group of patients was not significant. In the context of our study, it is crucial to note that not all of the proteins proved to be related to GFR (as RANTES and IL-12p70 did not show significant differences in concentration regarding GFR). What is more, there was a significant negative moderate correlation between GFR and MIG, MCP-1, and IL-8 and a significant negative weak correlation for IP-10, but not significant for RANTES and IL-12p70. Research by Molesh et al., who examined anxiety and depression factors in hemodialysis patients, showed that nearly 20% of patients experienced anxiety. They also found that the female gender was significantly associated with anxiety [44]. Shafi et al., using the Hospital Anxiety and Depression Scale (HADS), demonstrated that among the studied patients, the prevalence of anxiety was as high as 71.2%, regardless of disease progression. In contrast, moderate to severe anxiety was observed in 34.6% of the patients [45]. Cukor et al. also assessed anxiety disorders in hemodialysis patients, finding that 45.7% met the criteria for anxiety disorders [46]. As seen, anxiety is an inherent element of CKD and can intensify as the disease progresses. This group of patients not only faces anxiety disorders but also experiences reduced quality of life, often due to increased diagnostic and therapeutic rigor. Sharma et al. evaluated the health-related quality of life in CKD patients using the SF-36 and found that patients with CKD had a lower quality of life, which deteriorated as the disease progressed [47]. A meta-analysis by Fletcher et al. on the quality of life in CKD showed that quality of life was reduced in all CKD patients compared to those without CKD. In addition, the worst quality of life was observed in patients undergoing dialysis therapy. Post-transplant patients had a higher quality of life in comparison to patients with CKD but still lower than individuals without coexisting kidney diseases [48]. As evident from these studies, most patients with CKD and after kidney transplants have a lower health-related quality of life. Decreased quality of life and coexisting chronic anxiety can lead to depression and even PTSD (Post-Traumatic Stress Disorder) [2]. Therefore, it is crucial to identify biomarkers that can highlight patients, particularly those at risk of anxiety and reduced quality of life, allowing for more precise monitoring of their mental health and the provision of psychological and psychiatric care. In our study (which tested the differences between groups with the more conservative Bonferroni method) comparisons that were made between groups of patients with higher and lower levels of anxiety showed no significant difference in studied proteins levels. Correlations between all types of anxiety and the proteins were nonsignificant and very weak. RANTES levels proved to be statistically significant as a separate variable only in the logistic regression model of overall anxiety, but the chances of increasing STAI-total increased by only 0.1%. It should be borne in mind while taking their clinical implications under advisement. Although our study ultimately did not indicate significant differences in the protein levels we analyzed, the existing literature describes correlation between some of them and increased levels of anxiety. Camacho-Arroyo et al. evaluated the chemokine profile in pregnant women with symptoms of depression and anxiety. The Hamilton Anxiety Rating Scale and the Hamilton Depression Rating Scale were used to assess the patients. Their findings showed a positive correlation between RANTES levels and high anxiety levels (*p* < 0.05) compared to the control group. Among the chemokines evaluated in this study, the IL-8 levels were associated with high anxiety scores in the group of patients exhibiting moderate to severe anxiety [28]. Similar results were obtained by Ogłodek et al., who found elevated RANTES levels in the anxiety disorders group. Additionally, RANTES levels were significantly higher in women than in men [49]. Nonetheless, our study will undoubtedly help to avoid the file drawer effect that in the long-term leads to falsifying the scientific image of reality by erroneously treating statistically not significant results as irrelevant and of no value. However, our results cannot be evaluated without a profound insight into the scientific studies of other researchers and without reference to their findings. In our study, we also evaluated the relationship between proteins and quality of life. Our data indicated that in the group of patients with a lower quality of life, IL-8 level was significantly elevated compared to the group with a higher quality of life. Similar results were presented by Li et al., who evaluated the correlations between health-related quality of life and inflammatory cytokines. Their findings indicate that the strongest correlation was observed with IL-8 and that higher levels of this cytokine were associated with a poorer quality of life [50]. The correlations between quality of life and IL-8 and RANTES were also significant, albeit weak. Furthermore, if a woman had elevated RANTES levels, the odds of a lower quality of life increased by 150%. This is the important voice in the discussion on biomarkers of mental health and a precious guide for researchers to decide on the future directions of exploring the area in this particular topic; however, more studies are warranted. Possibly, considering other vital factors for a patient’s condition, these proteins could serve as some suggestion for clinicians to take a closer look at a patient’s mental health and advise some form of help from a psychotherapeutic or psychiatry specialist. Our analysis controverts the utility of the studied proteins as an anxiety biomarker but indicates that RANTES or IL-8 could serve as some kind of a hint for a clinician in a patient’s holistic care. Simultaneously, anxiety and health-related quality of life in chronically ill patients and kidney transplant recipients are definitely related, as shown not only by our study but also by other available publications. Both IL-8 and RANTES are directly linked to inflammation and elevated anxiety. Patients experiencing chronic anxiety report significantly reduced quality of life [47,48,51]. In conclusion, our study provides significant insights into the correlation between protein concentrations, anxiety levels, and reduced quality of life in patients with CKD and those who have undergone organ transplantation. However, certain limitations of this study must be acknowledged, as they may affect the interpretation of the results. Firstly, our research group consisted of only 107 participants, which may limit the possibility of generalizing the results. Although a power analysis indicated that the sample size was accurate to draw conclusions, it would be beneficial to conduct similar multicenter studies on a larger number of participants to obtain more representative data. Additionally, some patients were reluctant to complete all the questionnaires, which could have influenced their responses and the overall results. Moreover, the questionnaires were filled out during dialysis sessions, which might have significantly impacted the patients’ temporary anxiety levels due to the stress and discomfort associated with the treatment process. It is also worth noting that our research did not take into account other important factors, for instance: history of mental illnesses, emotional trauma, or the level of support from the family which could influence anxiety levels and quality of life in patients. The logistic regression models used in this study pose an attempt to capture a comprehensive view of the factors influencing quality of life and anxiety in CKD or transplant patients. The explanatory power of the logistic models suggests that other important factors were omitted. Although there is some relationship between inflammation and psychological well-being, other unexamined factors may play a larger role in determining these outcomes. Immunosuppressants proved to be important variables, which significantly elevated the explanatory power for SF-36, s-STAI, and STAI-total. Multicollinearity among cytokines is also a factor that can complicate the analyses, and more rigorous control of these interrelations is needed. Even though VIF values were essentially acceptable, there could exist other factors that could be confounders in this analysis. Methodological improvements and inclusion of other vital factors into analyses could increase the robustness of the results and provide a more accurate understanding of the interactions between inflammation, anxiety, and quality of life. Furthermore, the group of patients in our study was not randomly selected, which is an example of selection bias. The patients who filled out the questionnaires were informed that they were participating in a study, which might have affected their behavior, consciously or unconsciously, leading to observation bias. All of the above factors may impact the reliability and interpretation of the results, which should be considered when planning future research. In the future, conducting studies with a control group consisting of individuals with elevated anxiety levels but without accompanying chronic diseases could also provide additional valuable information. All of the abovementioned limiting factors have to be taken into account to interpret the study results in a proper way and with adequate caution. In summary, despite certain limitations, our study highlights the need for applying a holistic approach to patients with chronic kidney disease and those who have undergone organ transplantation. Further research on larger and more diverse groups of participants is necessary to better understand these complex relationships and to develop more effective strategies for supporting these patients.

## 4. Materials and Methods

### 4.1. Study Design, Participants, and Selection Criteria

The participants were women after a kidney transplantation or women with CKD treated by hemodialysis. The surveys were conducted using the STAI and SF-36 questionnaires given to every woman (in paper version, https://livingwellcnc.com/wp-content/documents/Self%20Evaluation%20Questionnaire.pdf accessed on 14 December 2024). Blood was collected from every patient. The concentration of IL-8, RANTES, MIG, MCP-1, IP-10, and IL-12p70 were measured. Additionally, morphology, creatinine, ALT, GFR, electrolytes, glucose, and the concentration of tacrolimus and/or cyclosporin A (if the patient was treated with those drugs) were measured in the group of patients with a kidney transplant. The reason for CKD and the date of transplantation were checked in the patient’s medical records. The type of diabetes was also noted. Participation in the study was voluntary. The study group consisted of 107 women—101 women from the Nephrological Clinic and 6 from the Dialysis Center. Women who did not answer every question in the questionnaire were excluded. The following were the study’s inclusion criteria: age 18 and over, being a woman, being a patient of the Nephrological Clinic or the Dialysis Center of University Clinical Hospital in Szczecin, having a kidney transplant or being treated by hemodialysis due to a CKD, and a willingness to be a participant in this research. The following were the exclusion criteria: age under 18 years old, and not having completed the questionnaire.

### 4.2. Instruments

Paper copies of two questionnaires, STAI (Form Y-1 and Form Y-2) and SF-36, were handed out to the patients. We used a Polish version of SF-36 and translated by a language expert STAI questionnaire (from the English to the Polish language). STAI is a 40 statements questionnaire, which is divided into two parts, equinumerous considering the number of sentences that respondents have to take a position on. We used Form Y-1 and Form Y-2. The first subscale (s-STAI) assesses anxiety as a state, which relates to a transient emotional state. The second subscale (t-STAI) assesses anxiety as a trait, which relates to a respondent’s stable and persistent tendency towards anxiety. A 4 point Likert scale is used to code the responses. The answers coded as 1–4 are “not at all/almost never”; “somewhat/sometimes”; “moderately so/often”; “very much so/almost always”—in the first/second part of the STAI questionnaire, respectively. The score range that one may obtain as a STAI-total score is 20–160 (80 points for 20 statements). The cut-off level in STAI-total was 94 points, with the results lower than 94 points classified as lower anxiety and results of at least 94 points as higher anxiety. In the case of s-STAI and t-STAI the dividing level was set on 46 points. Adapted to Polish conditions, SF-36 is an 11 questions (36 statements) questionnaire for subjective health assessment in which the outcome may be in the range of 0–171 points. Points represent the respondent’s struggles and complaints, so the lower the score, the higher the quality of life. A result of 65 points was the cut-off level. Results lower than 65 points were classified as higher quality of life and results of at least 65 as lower quality of life. Split-half reliability and Cronbach’s alpha coefficient were used to evaluate the two questionnaires’ reliability. The coefficient outcome is in the range of 0 to 1. If the reliability coefficient is larger than 0.6 and smaller than 1, the scale is considered reliable. The split-half for STAI was used to divide the STAI questionnaire into the first and second halves (s-STAI/Form Y-1 and t-STAI/Form Y-2) and SF-36 into two parts which were determined according to odd and even numbers. In both questionnaires, items were evaluated by the Spearman–Brown split-half coefficient.

### 4.3. Laboratory Analysis

We used the BD™ CBA Human Chemokine Kit (Fisher Scientific, Hampton, NH, USA), The BD™ CBA Human Inflammatory Cytokines Kit (Fisher Scientific, Hampton, NH, USA), and the cytometric method using BD FacsCalibur Flow Cytometer (BD Biosciences, San Jose, CA, USA) for analysis of specific chemokines in serum samples from patients with CKD or after KTx. The first kit can be used to quantitatively measure interleukin-8 (IL-8) and interleukin 12p70 (IL-12p70) protein levels. The second one can be used to quantitatively measure RANTES (CCL5/RANTES), Monokine-induced by Interferon-γ (CXCL9/MIG), Monocyte Chemoattractant Protein-1 (CCL2/MCP-1), and Interferon-γ-induced Protein-10 (CXCL10/IP-10) levels in a single sample. BD CBA assays provide a method of capturing a soluble analyte or set of analytes with beads of a known size and fluorescence, making it possible to detect analytes using flow cytometry. Each capture bead in the kit has been conjugated with a specific antibody. The detection reagent provided in the kit is a mixture of phycoerythrin (PE)-conjugated antibodies, which provides a fluorescent signal in proportion to the amount of bound analyte. When the capture beads and detector reagent are incubated with an unknown sample containing recognized analytes, sandwich complexes (capture bead + analyte + detection reagent) are formed. These complexes can be measured using flow cytometry to identify particles with fluorescence characteristics of both the bead and the detector. All flow cytometry results were analyzed using BD CellQuest Pro (Becton Dickinson, Franklin Lakes, NJ, USA) and FSC Express version 4.0 software (DeNovo).

### 4.4. Statistical Methods

JASP software was used to perform statistical analysis. Descriptive statistic methods were used in the beginning to obtain the basic data characteristics of the studied population: arithmetic mean, median, minimum and maximum value, and asymmetry coefficient (skewness). We applied a power analysis to determine if the sample size was adequate to detect significant effects [52]. The distribution of the protein concentrations was significantly different from a normal distribution, and the other variables were nominal or also did not have a normal distribution, therefore we used nonparametric significance tests: two-sided and one-sided Mann–Whitney U test (for two populations) in order to calculate comparisons [53]. To strengthen the analysis, a correction for multiple comparisons—the Bonferroni method—was applied [54]. In order to calculate sample correlations, the Spearman correlation coefficient was used [55]. A logistic regression model was used to isolate those variables that had a significant impact on increasing the odds of anxiety level increasing. Immunosuppressants, age, and GFR were included as variables in the regression models to control their impact on cytokine levels and psychological outcomes. The dependent variable in this model was dichotomous and the independent variables could be quantitative as well as qualitative [56]. To ensure the logistic models are not compromised, we applied a test for multicollinearity among independent variables using VIF (variance inflation factor) [57]. In the logistic regression model, to assess the degree of fit to empirical data, the measure called *R*^2^_*count*_ and receiver-operating characteristic (ROC) curves were obtained [58,59].

### 4.5. Ethics

Participation in the study was voluntary. The Declaration of Helsinki was followed in the conduct of the study. The study was approved by the university’s Ethics Committee on 20 December 2021 and given the ethical code number—KB-0012/54/2021.

## 5. Conclusions

Among all of the studied proteins, IL-8 and RANTES proved to have a relationship with the health-related quality of life of patients after kidney transplantation or currently being treated with dialysis. Other studied proteins showed mostly insignificant relationships with the mental health of patients. In our research, the studied proteins did not prove to be a good indicator of patients’ anxiety and the relationships with the anxiety were mostly insignificant. However, there was a significant moderate correlation between patients’ level of anxiety and quality of life. This study shows that special care should be provided to chronically ill women (especially with CKD or after KTx) with higher RANTES or IL-8 concentration. They would highly benefit from close monitoring of mental health, education in the area of methods for coping with stress and anxiety, or early interventions in case of possible depression. Professional help from a psychologist or even a psychiatrist could have a positive impact on their health. However, more studies on this topic are necessary.

## Figures and Tables

**Table 1:** Descriptive statistics of the participants of the study.

	Value
Minimum age Maximum age Average age	25 years 81 years 55.1 years
Median	57 years
Hemodialysis	6 patients
KTx	101 patients
DM	35/107 patients—34.6%
DM induced by KTx	24/35 patients—68.6%
DM1	5/35 patients—14.3%
DM2	6/35 patients—17.1%

**Table 2:** Descriptive statistics about the CKD in our patients.

	G1	G2	G3a	G3b	G4	G5	
Women	4	26	30	23	17	7	
	**GN**	**ADPKD**	**DM**	**HT**	**Unknown**		
Women	34	10	9	6	48		
% of women	31.78%	9.35%	8.41%	5.61%	44.86%		
	**TACR**	**MMF**	**ENC**	**CSA**	**EVE**	**AZA**	**SIR**
Women	77	68	59	12	8	8	2
% of women	76%	67%	58%	12%	8%	8%	2%

Note: GN—glomerulonephritis, ADPKD—autosomal dominant polycystic kidney disease, DM—diabetes mellitus, HT—hypertension, Unknown—unknown reason of the CKD in a patient, TACR—tacrolimus, MMF—mycophenolate mofetil, ENC—Encorton, CSA—cyclosporin A, EVE—Everolimus, AZA—azathioprine, SIR—sirolimus.

**Table 3:** Descriptive statistics of two questionnaires, SF-36 and STAI, for the studied population.

	SF-36	s-STAI	t-STAI	STAI-Total
Valid	107	107	107	107
Median	65.000	36.000	38.000	75.000
Mean	70.738	36.140	40.243	76.383
Std. Deviation	32.873	11.129	10.272	20.553
Skewness	0.525	0.725	0.327	0.490
Std. Error of Skewness	0.234	0.234	0.234	0.234
Minimum	9.000	20.000	20.000	40.000
Maximum	152.000	71.000	64.000	133.000
25th percentile	44.500	28.000	32.500	60.500
50th percentile	65.000	36.000	38.000	75.000
75th percentile	90.500	43.500	48.500	90.500

**Table 4:** Descriptive statistics of six studied proteins: Il-8, RANTES, MIG, MCP-1, IP-10, Il-12p70.

	IL-8	RANTES	MCP-1	IP-10	MIG	IL-12p70
Valid	107	107	107	107	107	107
Median	14.335	1916.950	15.950	81.850	91.830	3.530
Mean	22.935	1753.794	22.247	144.921	178.822	5.155
IQR	15.835	1126.640	19.650	90.550	95.240	3.255
Range	286.065	2076.680	145.660	1434.090	1936.110	35.410
Minimum	3.085	443.890	3.450	13.950	24.640	0.200
Maximum	289.150	2520.570	149.110	1448.040	1960.750	35.610

Note: The measurement unit for Median, Mean, IQR, Range, Minimum and Maximum is [ρg/mL].

**Table 5:** Correlation table.

Variable		s-STAI	t-STAI	STAI-Total	SF-36	Age	Years After KTx	GFR	IL-8	RANTES	MIG	MCP-1	IP-10	IL-12p70
1. s-STAI	Sperman’s rho	-												
	*p*-value	-												
2. t-STAI	Sperman’s rho	0.769	-											
	*p*-value	<0.001	-											
3. STAI-total	Sperman’s rho	0.458	0.940	-										
	*p*-value	<0.001	<0.001	-										
4. SF-36	Sperman’s rho	0.944	0.498	0.579	-									
	*p*-value	<0.001	<0.001	<0.001	-									
5. Age	Sperman’s rho	−0.014	−0.070	−0.039	0.277	-								
	*p*-value	0.886	0.473	0.693	0.004	-								
6. Years after KTx	Sperman’s rho	0.045	−0.035	8.755 × 10^−5^	0.073	0.174	-							
	*p*-value	0.654	0.727	0.999	0.466	0.083	-							
7. GFR	Sperman’s rho	−0.122	0.088	−0.002	−0.069	−0.073	0.054	-						
	*p*-value	0.211	0.368	0.983	0.479	0.452	0.594	-						
8. IL-8	Sperman’s rho	0.130	0.014	0.076	0.310	0.372	−0.172	−0.337	-					
	*p*-value	0.183	0.888	0.434	0.001	<0.001	0.085	<0.001	-					
9. RANTES	Sperman’s rho	0.024	0.004	0.005	0.241	0.136	0.051	0.053	0.237	-				
	*p*-value	0.808	0.966	0.957	0.012	0.162	0.613	0.588	0.014	-				
10. MIG	Sperman’s rho	0.019	−0.130	−0.064	0.064	0.423	−0.065	−0.443	0.489	0.023	-			
	*p*-value	0.847	0.181	0.514	0.510	<0.001	0.518	<0.001	<0.001	0.812	-			
11. MCP-1	Sperman’s rho	0.049	−0.089	−0.036	0.097	0.223	−0.212	−0.365	0.480	0.161	0.454	-		
	*p*-value	0.618	0.362	0.710	0.322	0.021	0.034	<0.001	<0.001	0.098	<0.001	-		
12. IP-10	Sperman’s rho	−0.046	−0.145	−0.110	−0.028	0.305	−0.104	−0.295	0.348	−0.097	0.693	0.431	-	
	*p*-value	0.639	0.135	0.258	0.773	0.001	0.300	0.002	<0.001	0.318	<0.001	<.001	-	
13. IL-12p70	Sperman’s rho	0.002	−0.053	−0.013	0.069	0.015	−0.076	−0.048	0.330	0.105	0.087	0.121	6.368 × 10^−4^	-
	*p*-value	0.980	0.589	0.898	0.478	0.878	0.448	0.622	<0.001	0.284	0.374	0.213	0.995	-

**Table 6:** Logistic regression model parameter evaluations and coefficients. Wald Test.

					Wald Test		
	Estimate	Standard Error	Odds Ratio	z	Wald Statistic	df	*p*
(Intercept)	0.388	0.674	1.474	0.576	0.331	1	0.565
RANTES	0.917	0.445	2.502	2.059	4.240	1	0.039
IL-8 (higher)	0.155	0.457	1.168	0.339	0.115	1	0.734
GFR (higher)	−0.458	0.473	0.632	−0.970	0.941	1	0.332
age	0.802	0.458	2.229	1.750	3.061	1	0.080
TAC	−1.309	0.553	0.270	−2.366	5.599	1	0.018
AZA	0.605	0.944	1.832	0.641	0.411	1	0.521
sirolimus	−17.214	1029.122	3.341 × 10^−8^	−0.017	2.798 × 10^−4^	1	0.987

Note: SF-36 level “higher” coded as class 1.

**Table 7:** Performance metrics of logit model for SF-36.

	Value
Accuracy AUC Sensitivity	0.664 0.740 0.611
Specificity	0.717

**Table 8:** Confusion matrix—classification accuracy of the logistic regression model.

Observed	Predicted Lower	Predicted Higher	% Correct
Lower	38	15	71.698
Higher Overall % Correct	21	33	61.111 66.355

Note: The cut-off value is set to 0.5.

**Table 9:** Multicollinearity diagnostics.

	Tolerance	VIF
TAC	0.948	1.055
AZA	0.906	1.103
GFR	0.859	1.164
RANTES	0.944	1.059
IL-8	0.894	1.119
Age	0.907	1.102
sirolimus	1.000	1.000

**Table 10:** Logit model parameter evaluations.

					Wald Test		
	Estimate	Standard Error	Odds Ratio	z	Wald Statistic	df	*p*
(Intercept)	−0.964	1.644	0.381	−0.587	0.344	1	0.558
RANTES	0.001	0.001	1.001	2.237	5.003	1	0.025
Age	−0.026	0.019	0.974	−1.382	1.910	1	0.167
GFR	−0.012	0.013	0.988	−0.920	0.846	1	0.358
CsA	−2.114	1.211	0.121	−1.746	3.049	1	0.081
AZA	2.425	1.138	11.308	2.132	4.547	1	0.033
TAC	−0.871	0.713	0.419	−1.220	1.489	1	0.222
MMF	−0.034	0.634	0.967	−0.054	0.003	1	0.957
PRED.	0.246	0.573	1.279	0.429	0.184	1	0.167

Note: STAI-total level “higher” coded as class 1.

**Table 11:** Performance metrics for logit model for STAI-total.

	Value
Accuracy	0.813
AUC Sensitivity Specificity	0.751 0.174 0.988

**Table 12:** Confusion matrix—classification accuracy of the logit model.

Observed	Predicted Lower	Predicted Higher	% Correct
Lower	83	1	98.810
Higher	19	4	17.391
Overall % Correct			81.308

Note: The cut-off value is set to 0.5.

**Table 13:** Multicollinearity diagnostics.

	Tolerance	VIF
RANTES	0.875	1.143
Age	0.888	1.126
GFR	0.760	1.316
CsA	0.522	1.914
AZA	0.583	1.715
TAC	0.584	1.712
MMF	0.668	1.496
Prednisolone	0.820	1.220

**Table 14:** Logit model parameter evaluations and coefficients.

					Wald Test		
	Estimate	Standard Error	Odds Ratio	z	Wald Statistic	df	*p*
(Intercept)	−2.701	2.174	0.067	−1.243	1.544	1	0.214
RANTES	0.001	0.001	1.001	1.617	2.617	1	0.106
MIG	0.002	0.001	1.002	1.885	3.554	1	0.059
Age	−0.022	0.024	0.978	−0.927	0.859	1	0.354
GFR	−0.004	0.016	0.996	−0.270	0.073	1	0.787
TAC	0.662	0.846	1.939	0.783	0.612	1	0.434
MMF	−0.777	0.764	0.460	−1.081	1.036	1	0.309
AZA	1.635	0.978	5.129	1.671	2.792	1	0.095
PRED	−0.744	0.668	0.475	−1.081	1.168	1	0.280

Note: s -STAI level “higher” coded as class 1.

**Table 15:** Performance metrics for logit model for s-STAI.

	Value
Accuracy	0.879
AUC Sensitivity Specificity	0.771 0.267 0.978

**Table 16:** Confusion matrix—classification accuracy of the logit model.

Observed	Predicted Lower	Predicted Higher	% Correct
lower	90	2	97.826
higher	11	4	26.667
Overall % Correct			87.850

Note: The cut-off value is set to 0.5.

**Table 17:** Multicollinearity diagnostics.

	Tolerance	VIF
RANTES	0.869	1.150
MIG	0.595	1.681
Age	0.858	1.166
GFR	0.652	1.534
TAC	0.593	1.687
MMF	0.687	1.456
AZA	0.759	1.318
Prednisolone	0.850	1.176

## Data Availability

All data were collected in the Department of Reconstructive Surgery and Gynecological Oncology, Pomeranian Medical University in Szczecin.
